# WHO global gonococcal antimicrobial surveillance programmes, 2019–22: a retrospective observational study

**DOI:** 10.1016/j.lanmic.2025.101181

**Published:** 2025-09-24

**Authors:** Magnus Unemo, Monica M Lahra, Michelle J Cole, Daniel Marcano Zamora, Susanne Jacobsson, Patricia Galarza, Irene Martin, Kristen M Kreisel, Marcelo Galas, Silvia Bertagnolio, Pilar Ramon-Pardo, Ismael Maatouk, Teodora Wi

**Affiliations:** WHO Collaborating Centre for Gonorrhoea and other STIs, Department of Laboratory Medicine, Faculty of Medicine and Health, Örebro University, Örebro, Sweden; Institute for Global Health, University College London, London, UK; WHO Collaborating Centre for Sexually Transmitted Infections and Antimicrobial Resistance, New South Wales Health Pathology, Microbiology, Randwick, NSW, Australia; UK Health Security Agency, London, UK; Surveillance, Evidence and Laboratory Strengthening, Antimicrobial Resistance Division, WHO, Geneva, Switzerland; WHO Collaborating Centre for Gonorrhoea and other STIs, Department of Laboratory Medicine, Faculty of Medicine and Health, Örebro University, Örebro, Sweden; WHO Collaborating Centre for Antimicrobial Resistance, National Institute of Infectious Diseases–ANLIS ‘Dr Carlos G Malbrán’, Buenos Aires, Argentina; Public Health Agency of Canada, National Microbiology Laboratory, Winnipeg, MB, Canada; Division of STD Prevention, Centers for Disease Control and Prevention, Atlanta, GA, USA; Antimicrobial Resistance Special Program, Communicable Diseases and Environmental Determinants of Health Departments, Pan American Health Organization, Washington, DC, USA; Surveillance, Evidence and Laboratory Strengthening, Antimicrobial Resistance Division, WHO, Geneva, Switzerland; Communicable Diseases and Environmental Determinants of Health Departments, Pan American Health Organization/WHO, Washington, DC, USA; Department of the Global HIV, Hepatitis and STI programmes, WHO, Geneva, Switzerland; Department of the Global HIV, Hepatitis and STI programmes, WHO, Geneva, Switzerland

## Abstract

**Background:**

Gonorrhoea and gonococcal antimicrobial resistance (AMR) remain global public health concerns, and enhanced quality-assured global surveillance of gonococcal AMR is imperative to inform management guidelines and public health policies. We aimed to describe the results of surveillance of gonococcal AMR conducted globally by WHO and discuss the actions needed to retain our ability to treat gonorrhoea.

**Methods:**

In this retrospective observational study, we present gonococcal AMR data reported to WHO by 77 countries between Jan 1, 2019, and Dec 31, 2022. Gonococcal isolates were tested for minimum inhibitory concentrations of one to four key antimicrobials (ceftriaxone, cefixime, azithromycin, and ciprofloxacin) in each country. We used breakpoints for resistance and decreased susceptibility to antimicrobials from the European Committee on Antimicrobial Susceptibility Testing or Clinical Laboratory and Standards Institute.

**Findings:**

29 (39%) of 75 participating countries reported at least one isolate with resistance or decreased susceptibility to ceftriaxone, 28 (50%) of 56 reported resistance or decreased susceptibility to cefixime, 58 (88%) of 66 reported resistance to azithromycin, and 74 (99%) of 75 reported resistance to ciprofloxacin. Globally, azithromycin resistance is increasing, as is resistance or decreased susceptibility to ceftriaxone and cefixime, especially in the WHO Western Pacific region. Resistance to ciprofloxacin remained very high globally. Since 2017–18, the numbers of reporting countries, examined isolates, and resistant isolates have increased. However, surveillance levels remain inadequate in central America and the Caribbean, eastern Europe, and the WHO African, Eastern Mediterranean, and South-East Asia regions.

**Interpretation:**

Global AMR surveillance conducted by WHO is expanding and, in selected countries, improving through standardisation and quality assurance, as well as implementation of extragenital sampling, test of cure, and whole-genome sequencing. This approach provides evidence-based data for management guidelines and public health policies. Improvements in prevention, early diagnosis, treatment of patients and their contacts, surveillance (of infection rates, AMR, treatment failures, and antimicrobial use), and antimicrobial stewardship are essential. WHO supports this work through several global action plans on AMR, new global gonorrhoea treatment recommendations, surveillance, and research.

**Funding:**

None.

## Introduction

Gonorrhoea, including its serious complications and sequelae that disproportionally affect women, remains a serious public health threat internationally. In 2020, WHO estimated that there were 82·4 million new cases of gonorrhoea in adults globally.^1^ It is a grave concern that clinical antimicrobial resistance (AMR) in *Neisseria gonorrhoeae* has evolved to all antimicrobials used for treatment of gonorrhoea to date.^2–6^ Ceftriaxone, an extended-spectrum cephalosporin (ESC), is the only remaining treatment option (in monotherapy or together with azithromycin) in most countries worldwide.^3,6^ However, resistance or decreased susceptibility to ceftriaxone and occasional ceftriaxone treatment failures have been verified in many countries worldwide.^2–12^ It is especially worrying that ceftriaxone-resistant clones, for example, FC428, are transmitted internationally^7^ and that strains with ceftriaxone resistance plus high-level azithromycin resistance have been identified since 2018 in several countries.^10–12^ WHO lists *N gonorrhoeae* among the priority AMR pathogens for human health, but the global magnitude of the AMR problem remains mostly unknown, especially in low-income and middle-income countries.^1–6^ Many currently spreading AMR gonococcal strains appear to have evolved in Asia and then spread internationally.^2–12^ Consequently, regular global surveillance of gonococcal AMR is essential, as strongly emphasised since 2012 by the WHO global action plan to control the spread and impact of gonococcal AMR.^13^

The WHO Gonococcal Antimicrobial Surveillance Programme (GASP),^4–6^ a collaborative global network of regional, national, and local reference laboratories, was designed to collect gonococcal AMR data in all WHO regions and collaborate with existing gonococcal AMR surveillance programmes internationally. These programmes include, for example, European GASP (Euro-GASP), the US Gonococcal Isolate Surveillance Project, GASP-Canada, Gonococcal Antimicrobial Susceptibility Surveillance Programme–Argentina, Brazil-GASP (within the SenGono Project), China Gonococcal Resistance Surveillance Programme, UK Gonococcal Resistance to Antimicrobials Surveillance Programme, and Australian Gonococcal Surveillance Programme.^4–6^ WHO’s GASP AMR data have, over several decades, informed revisions of global, regional, and national gonorrhoea treatment guidelines, as well as public health strategies and policies developed by WHO and other public health or clinical organisations. The WHO Global Antimicrobial Resistance and Use Surveillance System (GLASS) includes the WHO Enhanced GASP (EGASP)^8,12,14^ and further supports the WHO global action plans on both gonococcal AMR^13^ and AMR in all bacterial priority pathogens.^15^ Gonococcal AMR data collected through both WHO GASP and GLASS are compiled, curated, and collectively reported.

We aimed to describe WHO GASP and GLASS results for AMR from 77 countries worldwide during 2019–22 for comparison with 2017–18 data^6^ and discuss crucial international and national priorities needed to retain the ability to successfully treat gonorrhoea.

## Methods

### Study design

This retrospective observational study examined global gonococcal AMR data submitted to WHO from Jan 1, 2019, to Dec 31, 2022. 77 countries (appendix 1) reported AMR for one to four key antimicrobials for gonococcal isolates (≥5 isolates per year) to WHO GASP,^4–6^ WHO EGASP^8,12^ or WHO GLASS (n=75 for ceftriaxone, n=56 for cefixime, n=66 for azithromycin, n=75 for ciprofloxacin, and n=52 for all four antimicrobials; some countries reported to several of these WHO programmes). Gonococcal AMR results were then curated and deduplicated by WHO. Gonococcal AMR data from five additional countries during 2019–22, identified on PubMed using the terms “*Neisseria gonorrhoeae”* AND “antimicrobial” OR “antibiotic” from Jan 1, 2019, to Oct 1, 2024, were also included (appendix 1). Data from WHO GASP and GLASS from 2017 to 2018^6^ were used for comparison. No patient-identifiable information was available in the present study, and no ethical approval was required.

### Procedures

WHO GASP design, methodologies, and quality assurance procedures were summarised previously.^4–6,16^ Briefly, for WHO’s global gonococcal AMR surveillance, appropriate sampling schemes and methods, laboratory techniques, and quality assurance procedures are recommended in WHO’s 2012 surveillance standards, the WHO EGASP general protocol,^14^ and WHO’s 2013 manual for laboratory and point-of-care diagnosis of sexually transmitted infections. Countries vary substantially in their approaches to gonococcal AMR surveillance—eg, some test all gonococcal isolates through sentinel sites with a predefined sample size on a monthly basis, whereas others use a predefined sample size, with isolates collected continuously for several months until the sample size has been reached. Moreover, many countries conduct only passive and voluntary AMR surveillance based on submitted laboratory data or ad hoc surveys in one or several sentinel sites every 2–3 years. WHO’s GASP and EGASP aim to examine the minimum inhibitory concentrations ([MICs] in mg/L) of ceftriaxone, cefixime, azithromycin, and ciprofloxacin (using MIC gradient strip tests [eg, Etest] or the agar dilution method) in 100 or more gonococcal isolates, where available, per country per year. In this study, countries reporting fewer than five isolates per year were excluded. Reporting countries interpreted antimicrobial MICs using resistance breakpoints for ceftriaxone, cefixime, and ciprofloxacin or the epidemiological cutoff value for azithromycin (isolates with azithromycin MIC >1 mg/L are reported as resistant here) stated by the European Committee on Antimicrobial Susceptibility Testing (EUCAST, version 15.0) or the Clinical Laboratory and Standards Institute (CLSI). The EUCAST clinical resistance breakpoints are MIC >0·125 mg/L for ceftriaxone and cefixime and MIC >0·064 mg/L for ciprofloxacin. The CLSI clinical breakpoints are MIC >0·25 mg/L for non-susceptibility or decreased susceptibility to ceftriaxone, MIC >0·25 mg/L for decreased susceptibility to cefixime, and MIC >0·5 mg/L for resistance to ciprofloxacin. WHO recommends the use of the 2024 WHO gonococcal reference strains to facilitate the standardisation and quality control of gonococcal culture methods and assessment of antimicrobial MIC values and aid the comparability of WHO GASP data globally.^16^ These reference strains enable intralaboratory and interlaboratory comparison of results at local or national centres with variability in laboratory methods. In WHO’s GASP and GLASS, many countries report insufficient details regarding AMR testing methods, susceptibility or resistance breakpoints, and exact MICs. Additionally, the CLSI only recommends susceptibility breakpoints for ceftriaxone and cefixime. Accordingly, for ceftriaxone and cefixime, resistance and decreased susceptibility could only be reported combined. The primary outcome of this study was the numbers and percentages of isolates with resistance to azithromycin and ciprofloxacin and resistance or decreased susceptibility to ceftriaxone and cefixime.

### Statistical analysis

Data were analysed in Microsoft Excel (version 2408).

### Role of the funding source

There was no funding source for this study.

## Results

The numbers of countries from the six WHO regions (European region, region of the Americas, Western Pacific region, African region, Eastern Mediterranean region, and South-East Asian region) reporting gonococcal AMR data during 2019–22 are detailed in the table. The total numbers of isolates examined for any of the antimicrobials during 2019–22 ranged from 65 624 (cefixime) to 120 248 (ceftriaxone); in 2022 alone, the range was from 19 450 (cefixime) to 31 201 (ceftriaxone; appendix 1).

During 2019–22, 29 (39%) of 75 participating countries reported isolates with resistance or decreased susceptibility to ceftriaxone, 28 (50%) of 56 reported isolates with resistance or decreased susceptibility to cefixime, 58 (88%) of 66 reported azithromycin-resistant isolates, and 74 (99%) of 75 reported ciprofloxacin-resistant isolates (table). For comparison, in 2017–18, 21 (31%) of 68 countries reported isolates with resistance or decreased susceptibility to ceftriaxone, 24 (47%) of 51 reported isolates with resistance or decreased susceptibility to cefixime, 51 (84%) of 61 reported azithromycin-resistant isolates, and 70 (100%) of 70 reported ciprofloxacin-resistant isolates.^6^

Data from WHO GASP and GLASS for 2019–22 are in appendix 1. In 2022, 57 countries reported data on ceftriaxone resistance for five or more isolates. The number of isolates ranged from five (Bolivia) to 8199 (Australia), with 36 (63%) countries examining 100 isolates or more (appendix 1) compared with 29 (47%) of 62 in 2018.^6^ Overall, 17 (30%) of 57 countries reported isolates with resistance or decreased susceptibility compared with 17 (27%) of 62 in 2018.^6^ 45 countries reported cefixime data, with the number of isolates ranging from eight (Estonia) to 3855 (Canada). 32 (71%) countries tested 100 or more isolates compared with 29 (47%) of 62 in 2018.^6^ Overall, 16 (36%) of 45 countries reported isolates with resistance or decreased susceptibility to cefixime compared with 19 (39%) of 49 in 2018.^6^

**Figure 1:**
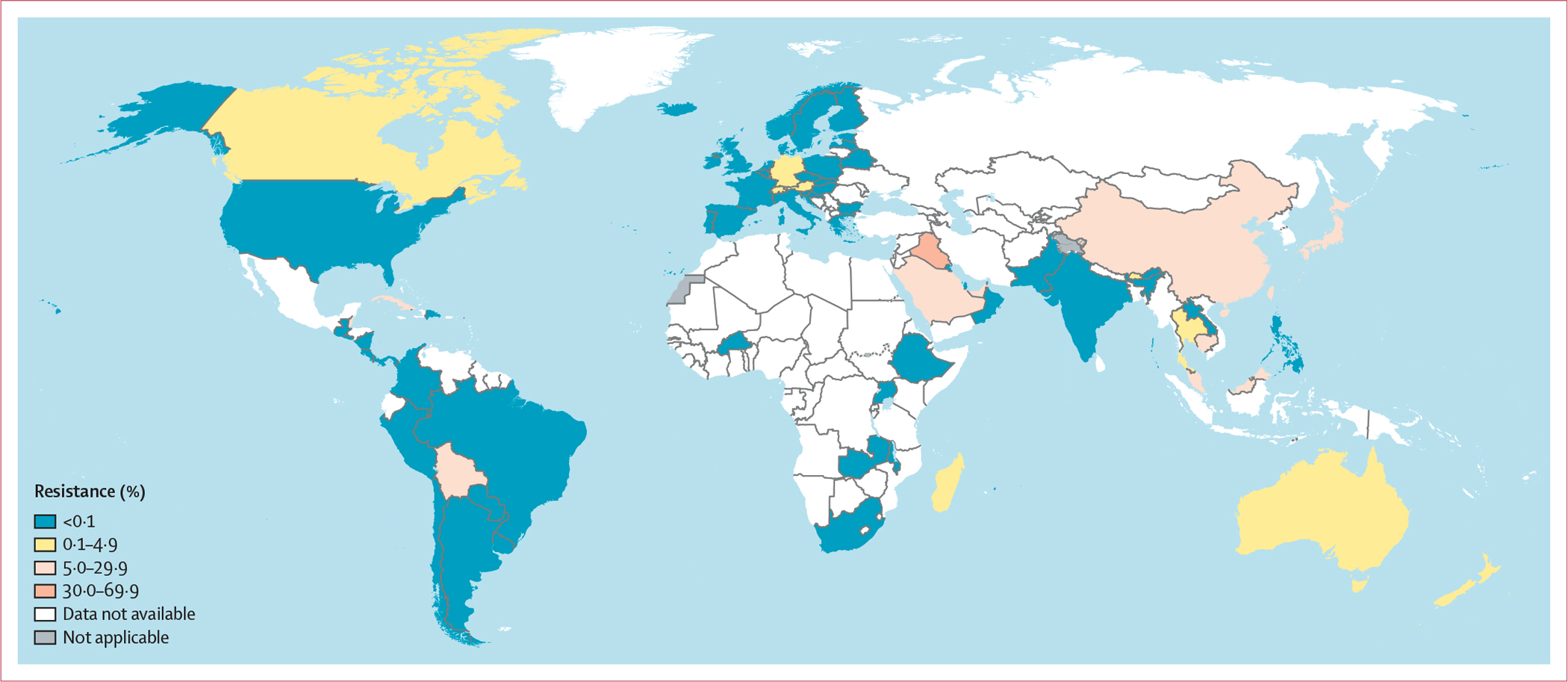
Percentage of *Neisseria gonorrhoeae* isolates with resistance or decreased susceptibility to ceftriaxone in 75 countries in 2022 Resistance in the figure key indicates resistance or decreased susceptibility. For Belarus, Burkina Faso, Croatia, Latvia, Madagascar, and Panama, data are from 2019 (<5 isolates per year reported during 2020–22). For Belize, Bhutan, Guatemala, Malawi, Switzerland, and Zambia, data are from 2020 (<5 isolates per year reported during 2021–22). For Brazil, Cuba, El Salvador, Laos, Malaysia, and Mauritius, data are from 2021 (<5 isolates per year reported during 2022). Dotted and dashed lines on maps represent approximate border lines for which there might not yet be full agreement. Disputed territories (Western Sahara, Jammu, and Kashmir) were not applicable, and no data were available from these regions. Due to the low number of isolates in several countries, antimicrobial resistance levels in these countries should be interpreted with great caution (appendix 1).

**Figure 2:**
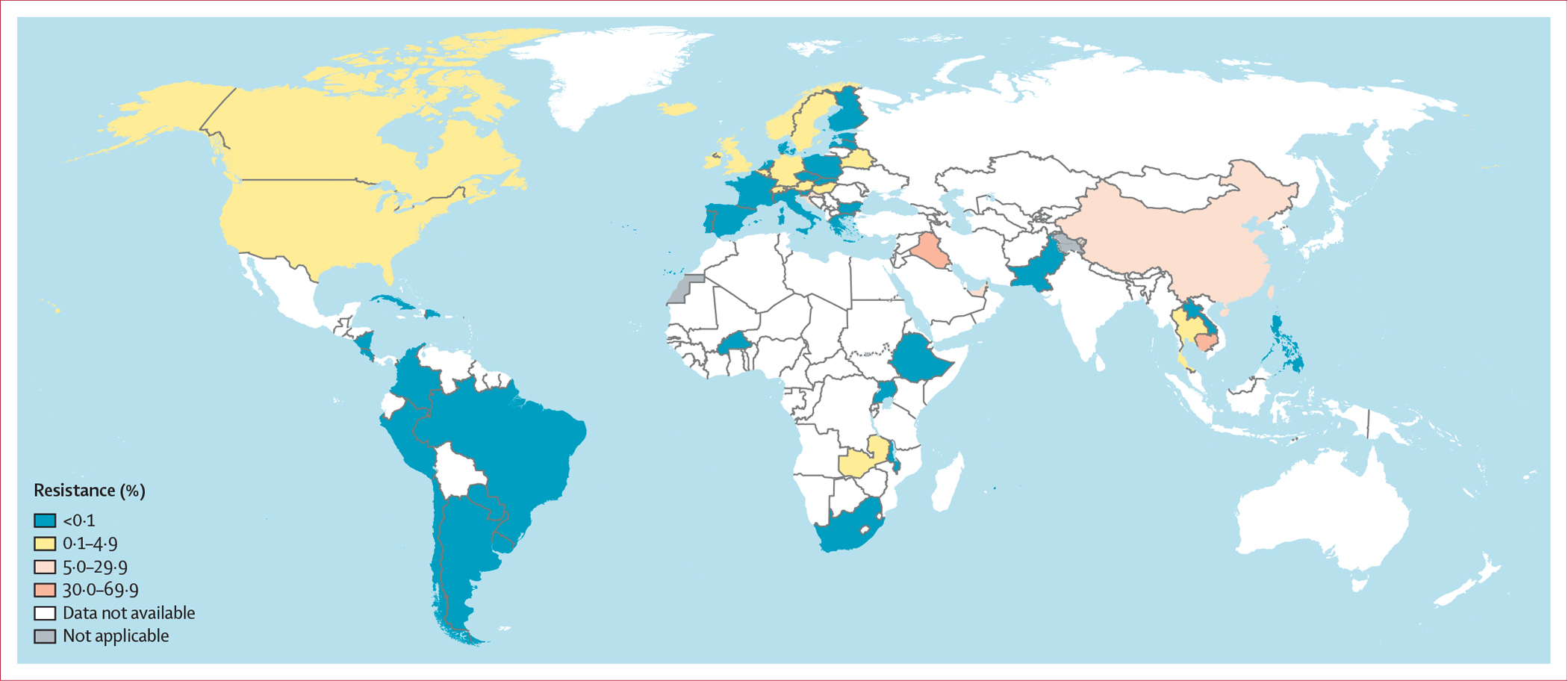
Percentage of *Neisseria gonorrhoeae* isolates with resistance or decreased susceptibility to cefixime in 56 countries in 2022 Resistance in the figure key indicates resistance or decreased susceptibility. For Belarus, Burkina Faso, Croatia, and Latvia, data are from 2019 (<5 isolates per year reported during 2020–22). For Malawi, Switzerland, and Zambia, data are from 2020 (<5 isolates per year reported during 2021–22). For Brazil, Cuba, Laos, and Mauritius, data are from 2021 (<5 isolates per year reported during 2022). Dotted and dashed lines on maps represent approximate border lines for which there might not yet be full agreement. Disputed territories (Western Sahara, Jammu, and Kashmir) were not applicable, and no data were available from these regions. Due to the low number of isolates in several countries, antimicrobial resistance levels in these countries should be interpreted with great caution (appendix 1).

4396 isolates from 23 EU or European Economic Area (EEA) countries in the WHO European region were examined in Euro-GASP in 2022.^17^ Using EUCAST clinical breakpoints, two (<1%) isolates were identified as resistant to ceftriaxone in 2022 (in Austria and Germany; figure 1), one (<1%) of 3541 was resistant in 2021, one (<1%) of 3291 was resistant in 2020, and three (<1%) of 4166 were resistant in 2019.^17^ In the UK, two (<1%) of 2620 isolates were reported to be resistant to ceftriaxone in 2022. Fewer than 1% to 2% of isolates were reported to be resistant to cefixime in eight (39%) of 23 EU or EEA countries reporting in 2022, but the overall prevalence of resistance was low, at less than 1% (15/4396 *vs* <1% [13/3531] in 2021, <1% [16/3290] in 2020, and 1% [39/4166] in 2019). The UK reported cefixime resistance in nine (1%) of 1290 isolates in 2022 (figure 2). In the WHO region of the Americas (9386 isolates), resistance or decreased susceptibility to ceftriaxone in fewer than 5% of isolates was reported in two (17%) of the 12 countries reporting these data in 2022 (11 [<1%] of 3855 isolates in Canada *vs* 15 [<1%] of 3123 in 2018^6^ and one [<1%] of 3684 in the USA *vs* nine [<1%] of 5160 in 2018^6^), and resistance or decreased susceptibility to ceftriaxone in more than 5% of isolates was reported in Bolivia (one [20%] of five; figure 1). All isolates in the other reporting countries were susceptible to ceftriaxone in 2022. Notably, resistance or decreased susceptibility to ceftriaxone was reported in Belize in 2020 (one [13%] of 8 isolates) and in Cuba in 2021 (one [20%] of five). Resistance or decreased susceptibility to cefixime was reported in 2022 in Canada (12 [<1%] of 3855 *vs* 17 [<1%] of 3122 in 2018^6^) and the USA (five [<1%] of 3684 *vs* 15 [<1%] of 5160 in 2018^6^). All isolates in the other nine reporting countries were susceptible to cefixime (figure 2). In the WHO Western Pacific region, resistance or decreased susceptibility to ceftriaxone was reported by seven (88%) of eight countries reporting AMR data for ceftriaxone (13 113 isolates) in 2022 compared with seven (70%) of ten in 2018.^6^ Three (38%) countries reported 5% or more isolates with resistance or decreased susceptibility to ceftriaxone (29 [18%] of 159 isolates reported in Cambodia, 227 [8%] of 2804 in China, and 15 [8%] of 190 in Japan), and four (50%) countries reported fewer than 5% isolates with resistance or decreased susceptibility to ceftriaxone (42 [1%] of 8199 isolates in Australia, one [4%] of 27 in Brunei, one [<1%] of 378 in New Zealand, and one [1%] of 186 in Singapore). In the Philippines, only ceftriaxone-susceptible isolates were reported in 2022 (figure 1). Notably, in Malaysia and Viet Nam,^8^ neither of which reported in 2022, more than 5% of isolates (five [6%] of 85 in Malaysia, in 2021, and 67 [27%] of 249 in Viet Nam in 2023 [the highest level of ceftriaxone resistance globally]) were reported to be resistant to ceftriaxone. Three (11%) of the 27 WHO Western Pacific region countries reported cefixime data in 2022 (3063 isolates). Cambodia and China reported more than 5% of isolates with resistance to cefixime (55 [35%] of 159 isolates in Cambodia and 449 [16%] of 2804 in China), whereas all isolates in the Philippines were susceptible (figure 2). Eight (38%) of 21 WHO Eastern Mediterranean region countries reported ceftriaxone data for 554 isolates in 2022. In Iraq, Saudi Arabia, and the United Arab Emirates, more than 5% of isolates were reported to have resistance or decreased susceptibility to ceftriaxone (seven [47%] of 15, one [6%] of 17, and 31 [10%] of 306, respectively), but all isolates in Bahrain, Kuwait, Oman, Pakistan, and Qatar were susceptible to ceftriaxone (figure 1). Only three (14%) WHO Eastern Mediterranean region countries reported cefixime data. Resistance or decreased susceptibility to cefixime was not found in Pakistan but was reported for more than 5% of isolates in Iraq (seven [64%] of 11) and the United Arab Emirates (26 [16%] of 161; figure 2). Only three (6%) of the 47 WHO African region countries provided ceftriaxone data in 2022 (812 isolates), and no isolates with resistance or decreased susceptibility were reported in Ethiopia, South Africa, or Uganda. Notably, no isolates with resistance or decreased susceptibility to ceftriaxone were reported in Mauritius in 2021, in Malawi or Zambia in 2020, or in Burkina Faso in 2019 (figure 1). For cefixime, no isolates with resistance or decreased susceptibility were reported in Ethiopia, South Africa, or Uganda in 2022, in Mauritius in 2021, in Malawi in 2020, or in Burkina Faso in 2019. However, in 2020, Zambia reported fewer than 5% of isolates with resistance or decreased susceptibility to cefixime (two [2%] of 122; figure 2). In the WHO South-East Asia region, only two [18%] of 11 countries reported ceftriaxone data (302 isolates) in 2022, with fewer than 5% of isolates reported to have resistance or decreased susceptibility in Thailand (one [<1%] of 253) and all isolates reported to be susceptible in India (figure 1). Notably, fewer than 5% of isolates were reported to have resistance or decreased susceptibility to ceftriaxone in India (one [3%] of 29) in 2021 and Bhutan (one [1%] of 97) in 2020. Only Thailand tested cefixime in 2022 (three [1%] of 243 isolates had resistance or decreased susceptibility; figure 2).

Azithromycin susceptibility data for at least five isolates were reported by 53 countries in 2022. The number of isolates ranged from five (in Bolivia and Nicaragua) to 8199 (in Australia), and 37 (70%) reporting countries examined 100 isolates or more (appendix 1) compared with 27 (47%) of 58 in 2018.^6^ Overall, 46 (87%) of 53 countries reported azithromycin-resistant isolates in 2022 compared with 44 (76%) of 58 in 2018.^6^ 36 (68%) of 53 countries reported resistance to azithromycin in at least 5% of isolates in 2022 compared with 40 (69%) of 58 in 2018.^6^

**Figure 3:**
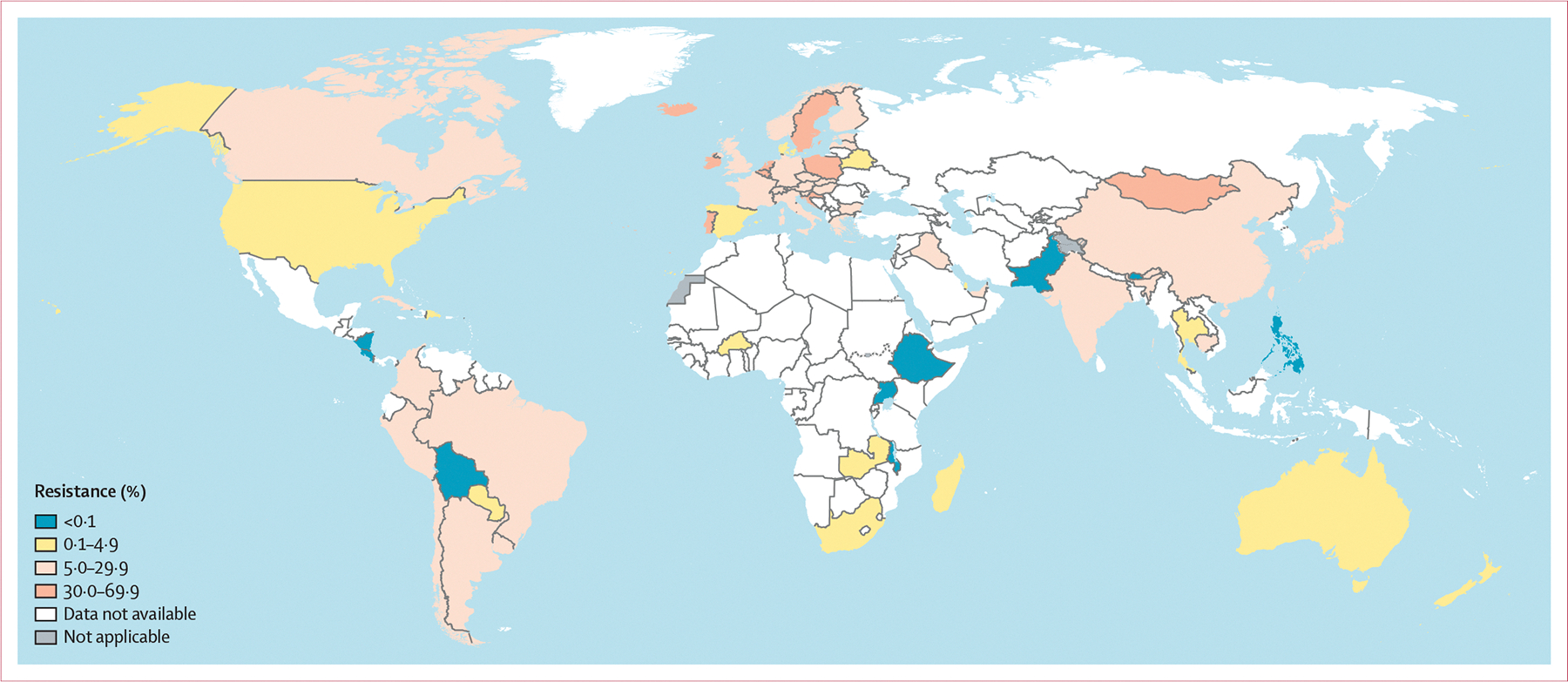
Percentage of *Neisseria gonorrhoeae* isolates with resistance to azithromycin in 66 countries in 2022 For Belarus, Burkina Faso, Croatia, Latvia, and Madagascar, data are from 2019 (<5 isolates per year reported during 2020–22). For Bhutan, Brazil, Malawi, Switzerland, and Zambia, data are from 2020 (<5 isolates per year reported during 2021–22). For Cuba and Mongolia, data are from 2021 (<5 isolates per year reported during 2022). Dotted and dashed lines on maps represent approximate border lines for which there might not yet be full agreement. Disputed territories (Western Sahara, Jammu, and Kashmir) were not applicable, and no data were available from these regions. Due to the low number of isolates in several countries, antimicrobial resistance levels in these countries should be interpreted with great caution (appendix 1).

As assessed with the EUCAST epidemiological cutoff value, azithromycin-resistant isolates were reported in all 23 (100%) Euro-GASP countries in the WHO European region in 2022 (4396 isolates; 21 [91%] countries reported resistance in ≥5% of isolates)^17^ compared with 24 (89%) of 27 in 2018.^6^ The UK also reported azithromycin resistance in more than 5% of isolates (figure 3). In the WHO region of the Americas, azithromycin resistance was found in nine (75%) of 12 countries reporting in 2022 (9509 isolates). Six (50%) countries (Argentina, Canada, Chile, Colombia, Peru, and Uruguay) reported resistance in at least 5% of isolates, whereas three (25%) countries (Dominican Republic, Paraguay, and the USA) reported resistance in fewer than 5% of isolates. In the WHO Western Pacific region, seven (88%) of eight reporting countries detected azithromycin resistance in 2022 (13 131 isolates) compared with five (71%) of seven in 2018.^6^ Five (63%) countries (Brunei, Cambodia, China, Japan, and Singapore) reported resistance in at least 5% of isolates, and two (25%) countries (Australia and New Zealand) reported resistance in fewer than 5% of isolates. All isolates in the Philippines were susceptible to azithromycin. In the WHO Eastern Mediterranean region (269 isolates), azithromycin resistance was reported in at least 5% of isolates in Iraq and the United Arab Emirates in 2022, whereas resistance in fewer than 5% of isolates was reported in Qatar. All isolates in Pakistan were susceptible to azithromycin. In the WHO African region, azithromycin resistance was detected in one (33%) of the three countries reporting in 2022 (809 isolates). South Africa reported resistance in fewer than 5% of isolates, but all isolates in Ethiopia and Uganda were susceptible to azithromycin. In the WHO South-East Asian region, azithromycin resistance was detected in both countries reporting in 2022 (302 isolates). India reported resistance in at least 5% of isolates, and Thailand reported resistance in fewer than 5% of isolates (figure 3).

**Figure 4:**
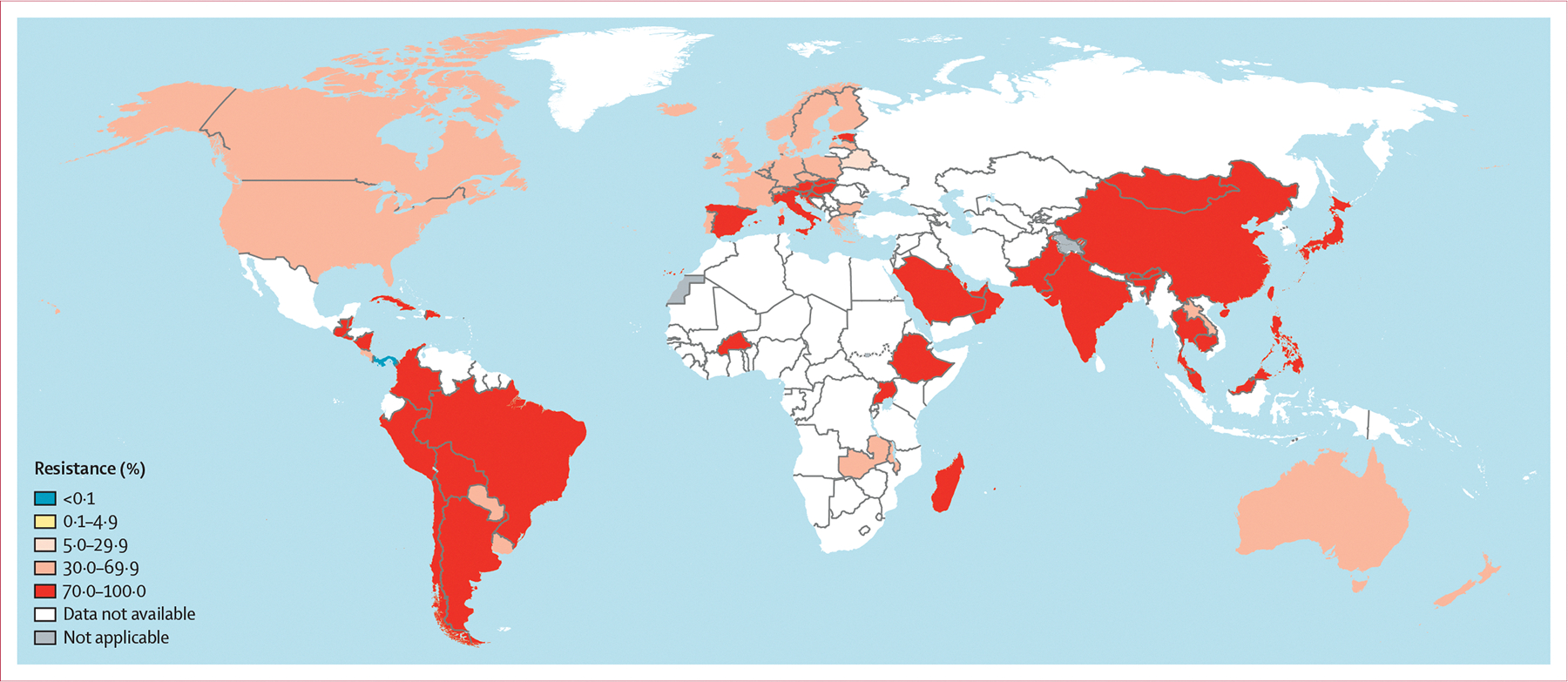
Percentage of *Neisseria gonorrhoeae* isolates with resistance to ciprofloxacin in 75 countries in 2022 For Belarus, Burkina Faso, Croatia, Latvia, Madagascar, Malawi, and Panama, data are from 2019 (<5 isolates per year reported during 2020–22). For Belize, Bhutan, Guatemala, Switzerland, and Zambia, data are from 2020 (<5 isolates per year reported during 2021–22). For Brazil, Cuba, El Salvador, Kosovo, Laos, Malaysia, Mauritius, and Mongolia, data are from 2021 (<5 isolates per year reported during 2022). Dotted and dashed lines on maps represent approximate border lines for which there might not yet be full agreement. Disputed territories (Western Sahara, Jammu, and Kashmir) were not applicable, and no data were available from these regions. Due to the low number of isolates in several countries, antimicrobial resistance levels in these countries should be interpreted with great caution (appendix 1).

For ciprofloxacin, 55 countries reported data for at least five isolates in 2022. The number of isolates ranged from five (in Bolivia) to 8199 (in Australia), and 35 (64%) reporting countries tested at least 100 isolates (appendix 1) compared with 29 (44%) of 66 in 2018.^6^ In 2022, all countries reported ciprofloxacin-resistant isolates, with numbers and percentages of resistant isolates ranging from 44 (33%) of 135 in Denmark to 36 (100%) of 36 in Bahrain. 12 (22%) countries in five WHO regions (Bahrain, Cambodia, China, Ethiopia, India, Oman, Pakistan, Peru, the Philippines, Saudi Arabia, Thailand, and Uganda) reported resistance to ciprofloxacin in at least 90% of isolates. In 39 (71%) countries, 50–90% of isolates were resistant to ciprofloxacin (figure 4).

## Discussion

In 2019–22, WHO GASP and GLASS identified that, globally, resistance to azithromycin and resistance or decreased susceptibility to ceftriaxone and cefixime had increased since 2017–18^6^ (both in terms of number of countries reporting at least one isolate with resistance or with resistance or decreased susceptibility and of total number of isolates with resistance or with resistance or decreased susceptibility), especially in the WHO Western Pacific region. These findings are in accordance with many other local, national, or international studies.^3,7–9,12,18^ Resistance to ciprofloxacin remains very high globally. An increasing number of countries are submitting susceptibility data and reporting the identification of isolates with resistance or decreased susceptibility to antimicrobials, and the total number of examined isolates is increasing. However, substantial concerns remain, including the suboptimal numbers of countries reporting and isolates examined in many WHO regions and the absence of epidemiological and clinical data, standardisation, and harmonised quality assurance, which limits the comparability of AMR data. Many countries with high prevalence of gonorrhoea but low diagnostic capacity and unrestricted access to antimicrobials still do not have robust surveillance, which results in opportunities for the emergence and spread of gonococcal AMR.^1–6^ Furthermore, gonococcal AMR surveillance remains especially inadequate in central America and the Caribbean, eastern Europe, and the WHO African, Eastern Mediterranean, and South-East Asian regions. Accordingly, to expand, improve, standardise, and quality assure global gonococcal AMR surveillance, WHO is substantially expanding EGASP, which focuses on selected countries in the different WHO regions. EGASP was initiated in Thailand in 2015 and in the Philippines in 2018, with Cambodia^12^ included in 2021, South Africa and Uganda in 2022, and Malawi, Indonesia, Zimbabwe, and Viet Nam in 2023;^8^ India, Brazil, Argentina, Côte d’Ivoire, and Qatar were included in 2024 and are currently at different stages of implementation. EGASP uses standardised and quality-assured protocols (including standardised internal controls, an external quality assessment, and WHO gonococcal reference strains^16^) and collects the demographic, behavioural, and clinical (including receipt of treatment) data of patients with gonorrhoea. EGASP has also implemented whole-genome sequencing,^8,16^ extragenital sampling, and test of cure. Of note, EGASP has additionally initiated phenotypic and genomic studies examining gonococcal susceptibility to the novel antimicrobials zoliflodacin^19^ and gepotidacin^20^ as well as a tetracycline susceptibility study to estimate the potential effect of doxycycline post-exposure prophylaxis^21^ on incident gonorrhoea cases in EGASP countries.

Appropriate and timely global surveillance data on gonococcal AMR are essential to refine international and national gonorrhoea treatment guidelines.^3,6^ In fact, use of WHO EGASP data has already resulted in a revision of Cambodia’s national gonorrhoea treatment guidelines,^12^ and Indonesia, the Philippines, South Africa, and Viet Nam^8^ are currently making revisions of their national treatment guidelines. These revisions will also align their national treatment guidelines with WHO’s new global gonorrhoea treatment guidelines, which recommend ceftriaxone 1 g monotherapy, in agreement with many other international and national treatment guidelines.^3^ According to the results of treatment of occasional ceftriaxone-resistant cases^5,7,22^ and pharmacodynamic studies that used a hollow fibre infection model for gonorrhoea,^23^ ceftriaxone 1 g can cure most urogenital infections caused by currently circulating ceftriaxone-resistant gonococcal strains (mostly with ceftriaxone MIC of 0·25–0·5 mg/L). However, oropharyngeal gonorrhoea cases caused by these ceftriaxone-resistant strains are more difficult to cure than urogenital infections, even with ceftriaxone 1 g.^5,7,22,23^ In fact, oropharyngeal gonorrhoea cases are generally more difficult to cure than urogenital and anorectal infections with most antimicrobials; moreover, oropharyngeal gonorrhoea is mostly asymptomatic and, therefore, difficult to detect without testing, and AMR determinants can be acquired from non-gonococcal *Neisseria* species.^2–5,7,22,23^ Notably, a multidose ceftriaxone or ESC regimen should not be excluded as salvage therapy. Furthermore, despite its limitations in treating oropharyngeal gonorrhoea using monotherapy, it would be valuable to have spectinomycin 2 g more widely available globally.^24^

Novel antimicrobials for the treatment of gonorrhoea caused by ceftriaxone-resistant gonococcal strains are imperative. Recently, the oral drugs zoliflodacin^19^ and gepotidacin^20^ showed non-inferiority in phase 3 randomised controlled clinical trials comparing them with ceftriaxone plus azithromycin therapy in the treatment of uncomplicated urogenital gonorrhoea. The main characteristics of zoliflodacin and gepotidacin are summarised in appendix 2 (p 1). However, occasional clinical or selected zoliflodacin-resistant and gepotidacin-resistant gonococcal isolates have been found.^25–29^ To enhance surveillance and support the licensing of zoliflodacin and gepotidacin for the treatment of uncomplicated gonorrhoea, WHO EGASP has initiated in-vitro phenotypic and genomic studies on these antimicrobials. Notably, lefamulin has also shown promising in-vitro efficacy against *N gonorrhoeae*,^30^ but evaluation of its pharmacodynamics (based on hollow fibre infection model studies) suggested that this drug is suboptimal for the treatment of gonorrhoea, and there are no clinical data regarding gonorrhoea treatment.^31^ Other new antimicrobials such as solithromycin and delafloxacin, which appeared promising on the basis of MIC data, have recently failed in their phase 3 clinical trials.^3^ Accordingly, although the gonococcal MICs of many new antimicrobials and other compounds show a high gonococcal activity in vitro, appropriate pharmacokinetic–pharmacodynamic data (eg, from mouse models or hollow fibre infection models that were used to inform dosing in the zoliflodacin and gepotidacin phase 3 clinical trials^23,26,31–33^) and clinical data for the treatment of both anogenital and oropharyngeal gonorrhoea are essential.

The main limitations of WHO GASP and GLASS^4–6^ include the suboptimal number of included countries in some WHO regions; the lack of harmonised and standardised sampling schemes, methods, and quality assurance; the low number and poor representativeness of isolates in many countries; the use of both EUCAST and CLSI breakpoints, which have minor differences; and the absence of epidemiological data for the patients with gonorrhoea. Despite these limitations, WHO GASP^4–6^ and WHO EGASP^8,12,14,18^ are the only large global gonococcal AMR surveillance programmes, annually including more than 70 countries, and the data from these programmes have informed many revisions of international and national treatment guidelines, as well as public health action and policies. Furthermore, WHO and the liaised GASPs are continuously working on improvements through advocacy, regular training of health-care workers and laboratory staff in GASP and GLASS methodologies, provision of WHO gonococcal reference strains for quality assurance and quality control of culture diagnostics and AMR testing,^16^ and inclusion of additional countries in WHO EGASP.^8,12^

In conclusion, resistance to azithromycin and resistance or decreased susceptibility to ceftriaxone and cefixime have increased globally, and resistance to ciprofloxacin has remained very high. Global surveillance of gonococcal AMR conducted by WHO through GASP^4–6^ and, in particular, WHO EGASP^8,12^ and GLASS, is expanding and improving, promoting standardisation, quality assurance, and implementation of extragenital sampling, test of cure, and whole-genome sequencing in selected countries. This surveillance provides evidence-based AMR data for the refinement of international and national gonorrhoea management guidelines and public health policies, including prescription policies and regulations. Improvements in prevention, early diagnosis, treatment of patients and their contacts, surveillance (of infection rates, AMR, treatment failures, and antimicrobial use), and antimicrobial stewardship are essential. Rapid, accurate, and affordable point-of-care diagnostic tests (detecting *N gonorrhoeae* and predicting its antimicrobial resistance or susceptibility), novel antimicrobials (eg, zoliflodacin^19^ and gepotidacin^20^), and vaccines for gonorrhoea^34^ are imperative. WHO supports this work through its global action plan to control the spread and impact of gonococcal AMR^13^ and global action plan on AMR,^15^ as well as its integrated global action plan for drug-resistant HIV, viral hepatitis, and sexually transmitted infections (under development), new global gonorrhoea treatment recommendations, surveillance, and research.

## Supplementary Material

Appendix 1

Appendix 2

## Figures and Tables

**Table:** WHO regions and countries reporting *Neisseria gonorrhoeae* isolates with resistance or decreased susceptibility to ceftriaxone and cefixime, respectively, and resistance to azithromycin and ciprofloxacin in 2019–22

	African (n=47)	Americas (n=35)	Eastern Mediterranean (n=21)	Europe (n=53)	South-East Asia (n=11)	Western Pacific (n=27)	Total (n=194)

**Ceftriaxone** *							
Countries reporting	8 (17%)	18 (51%)	8 (38%)	28 (53%)	3 (27%)	10 (37%)	75 (39%)
≥5% decreased susceptibility and resistance†	0	3/18 (17%)	3/8 (38%)	0	0	4/10 (40%)	10/75 (13%)
<5% decreased susceptibility and resistance	1/8 (13%)	2/18 (11%)	1/8 (13%)	8/28 (29%)	3/3 (100%)	4/10 (40%)	19/75 (25%)
Susceptibility	7/8 (88%)	13/18 (72%)	4/8 (50%)	20/28 (71%)	0	2/10 (20%)	46/75 (61%)
**Cefixime** *							
Countries reporting	7 (15%)	13 (37%)	3 (14%)	28 (53%)	1 (9%)	4 (15%)	56 (29%)
≥5% decreased susceptibility and resistance†	0	0	2/3 (67%)	1/28 (4%)	0	2/4 (50%)	5/56 (9%)
<5% decreased susceptibility and resistance	3/7 (43%)	2/13 (15%)	0	17/28 (61%)	1/1 (100%)	0	23/56 (41%)
Susceptibility	4/7 (57%)	11/13 (85%)	1/3 (33%)	10/28 (36%)	0	2/4 (50%)	28/56 (50%)
**Azithromycin**							
Countries reporting	7 (15%)	14 (40%)	5 (24%)	28 (53%)	3 (27%)	9 (33%)	66 (34%)
≥5% resistance†	0	10/14 (78%)	2/5 (40%)	27/28 (96%)	1/3 (33%)	7/9 (78%)	47/66 (71%)
<5% resistance	4/7 (57%)	2/14 (11%)	1/5 (20%)	1/28 (4%)	1/3 (33%)	2/9 (22%)	11/66 (17%)
Susceptibility	3/7 (43%)	2/14 (11%)	2/5 (40%)	0	1/3 (33%)	0	8/66 (12%)
**Ciprofloxacin**							
Countries reporting	7 (15%)	18 (51%)	7 (33%)	29 (55%)	3 (27%)	11 (41%)	75 (39%)
>90% resistance	5/7 (71%)	3/18 (17%)	5/7 (71%)	1/29 (3%)	3/3 (100%)	4/11 (36%)	21/75 (28%)
≥5–90% resistance†	2/7 (29%)	14/18 (78%)	2/7 (29%)	28/29 (97%)	0	7/11 (64%)	53/75 (71%)
<5% resistance	0	0	0	0	0	0	0
Susceptibility	0	1/18 (6%)	0	0	0	0	1/75 (1%)

Data are n (%) or n/N (%). Included countries reported at least one gonococcal isolate with resistance or decreased susceptibility to the specific antimicrobial.

* Resistance and decreased susceptibility were combined for ceftriaxone and cefixime due to the different antimicrobial resistance testing methods (minimum inhibitory concentration assessment with agar dilution or gradient strip tests) and breakpoints used, and because the Clinical Laboratory and Standards Institute recommends only susceptibility breakpoints for these antimicrobials.

† Resistance level for which WHO recommends discontinuing a first-line empirical antimicrobial regimen in gonorrhoea monotherapy.

## Data Availability

All data collected and analysed in this study are included in the main Article or appendix 1.

## Research in context

### Evidence before this study

Gonorrhoea is a major global public health concern. Antimicrobial resistance (AMR) in *Neisseria gonorrhoeae* is compromising the management and control of gonorrhoea, and it is feared that gonorrhoea might become untreatable due to resistance to all antimicrobials introduced for treatment. During recent years, reports from several countries have described resistance to the last remaining option for empirical first-line treatment—the third-generation cephalosporin ceftriaxone. Global surveillance of gonococcal AMR has been declared essential by WHO and other international and national public health organisations. We searched PubMed using the terms “*Neisseria gonorrhoeae”* AND “surveillance” AND “global” AND “antimicrobial susceptibility” OR “antimicrobial resistance” for full-length articles in English published from May 26, 1978, to Jan 1, 2024. Only three published original articles, all from WHO, have surveyed global gonococcal AMR. The most recent AMR data in these published papers were from 2018, and the main conclusions of these papers included the need for global gonococcal AMR surveillance to be substantially expanded (ie, increased number of countries and gonococcal isolates examined) and improved, with more quality assurance.

### Added value of this study

This study provides global gonococcal AMR data (for ceftriaxone, cefixime, azithromycin, and ciprofloxacin) from 2019 to 2022. A large number of countries (n=77) reported data, and the total numbers of isolates examined for any of the antimicrobials varied from 65 624 (cefixime) to 120 248 (ceftriaxone); in 2022 alone, the range was from 19 450 (cefixime) to 31 201 (ceftriaxone). We provide evidence that global azithromycin resistance is increasing, as is resistance or decreased susceptibility to ceftriaxone and cefixime, especially in the WHO Western Pacific region. Furthermore, in selected countries and different WHO regions, WHO has implemented standardised and quality-assured gonococcal AMR surveillance that also collects demographic, clinical, and behavioural data of patients and includes whole-genome sequencing of gonococcal isolates. Finally, the Article discusses essential actions to retain the ability to treat gonorrhoea, including new treatments, and summarises the main characteristics of the two new oral antimicrobials zoliflodacin and gepotidacin, which recently showed non-inferiority compared with a ceftriaxone plus azithromycin regimen for the treatment of uncomplicated urogenital gonorrhoea in phase 3 randomised controlled clinical trials.

### Implications of all the available evidence

Gonorrhoea could become untreatable due to high AMR levels. Resistance to the last remaining option for empirical first-line gonorrhoea treatment, ceftriaxone, is increasing internationally. Ceftriaxone resistance has already reached high levels in several countries in the WHO Western Pacific region, such as Cambodia, China, and Viet Nam, and ceftriaxone-resistant gonococcal strains are spreading globally. These findings are in accordance with many other local or national studies. Accordingly, further enhanced global gonococcal AMR surveillance is essential to monitor AMR trends, identify emerging AMR, and inform revisions of global, international, and national clinical management guidelines and public health policies. WHO has expanded and improved the quality of its global gonococcal AMR surveillance; however, there is inadequate surveillance in central America and the Caribbean or eastern Europe or in the WHO African, Eastern Mediterranean, and South-East Asia regions. Key priorities essential for effective future management and control of gonorrhoea should be incorporated into international and national action or response plans. These priorities include improving prevention, early diagnosis, and treatment of patients and their sexual contacts and enhancing surveillance (for infections, AMR, treatment failures, and antimicrobial use) and antimicrobial stewardship. Furthermore, rapid, accurate, and affordable point-of-care diagnostic tests, novel antimicrobials (eg, zoliflodacin and gepotidacin), and vaccines for gonorrhoea are imperative. WHO supports this work through several global action plans on AMR, new global gonorrhoea treatment recommendations, surveillance, and research.
