# Supplementary material for: WHO global gonococcal antimicrobial surveillance programmes, 2019–22: a retrospective observational study
Source: Lancet Microbe. Author manuscript; Available in PMC 2025 Dec 19. (PMC12715682; doi:10.1016/j.lanmic.2025.101181)
Supplement: Appendix 2 [file NIHMS2126708-supplement-Appendix_2.pdf]

THE LANCET
Microbe

Supplementary appendix 2

This appendix formed part of the original submission and has been peer reviewed.
We post it as supplied by the authors.

Supplement to: Unemo M, Lahra MM, Cole MJ, et al. WHO global gonococcal antimicrobial surveillance programmes, 2019–22: a retrospective observational study. *Lancet Microbe* 2025. https://doi.org/10.1016/j.lanmic.2025.101181

Table S2: **Main characteristics of the novel oral antimicrobials zoliflodacin[1] and gepotidacin[2,3] for the treatment of uncomplicated urogenital gonorrhoea**

**Antimicrobial (previous names)**	**Class**	**Mode of action**	**Bacterial target (mutations known to increase the MIC)**	**In-vitro activity against *N. gonorrhoeae***		**Phase 2 clinical trial[1,2]**	**Phase 3 clinical trial[3]**
				**MIC range**	**MIC90**		
Zoliflodacin (AZD0914, ETX0914)	Spiropyrimidinetrione (topoisomerase II inhibitor)	DNA biosynthesis inhibition and accumulation of double-strand cleavages[4,5]	DNA gyrase (GyrB) (GyrB D429N, D429A, D429V and K450T)[6-13]	≤0·002–8 mg/L[8,12-19]	0·125–0·25 mg/L[7,13-21]	***Oral dose*** 2 g×1 or 3 g×1[1]	***Oral dose*** 3 g×1
						Microbiological cure Genital: 98·0% (48/49) and 100% (47/47), respectively[1]	***Results (genital)*** Non-inferior to CRO 500 mg×1 + AZM 1 g×1 (-5·3% (95% CI 1·4, 8·7%) difference in cure)[a]
Gepotidacin (GSK2140944)	Triazaacenaphthylene (topoisomerase II inhibitor)	Inhibits DNA replication through interactions with GyrA (subunit of DNA gyrase) and ParC (subunit of topoisomerase IV)[22,23]	DNA gyrase (GyrA) and topoisomerase IV (ParC) (GyrA A92T, ParC D86N)[24-27]	≤0·015–>32 mg/L[24-26]	0·5 mg/L[24-26]	***Oral dose*** 1·5 g×1 or 3 g×1[2]	***Oral dose*** 3 g×1 + 3 g×1, 10-12 h apart[3]
						Microbiological cure Genital: 97% (29/30) and 95% (37/39), respectively[2]	***Microbiological cure*** Genital: 100% (187/187), Rectal: 100% (26/26), Pharyngeal: 87·5% (14/16)[3]

MIC=minimum inhibitory concentration. MIC90=MIC required to inhibit 90% of gonococcal strains. ×1=single dose. CRO=ceftriaxone. AZM=azithromycin. CI=confidence interval.

[a] Full results will soon be published.

References

1. Taylor SN, Marrazzo J, Batteiger BE, et al. Single-dose zoliflodacin (ETX0914) for treatment of urogenital gonorrhea. *N Engl J Med* 2018; **379**: 1835-45.
2. Taylor SN, Morris DH, Avery AK, et al. Gepotidacin for the treatment of uncomplicated urogenital gonorrhea: a phase 2, randomized, dose-ranging, single-oral dose evaluation. *Clin Infect Dis* 2018; **67**: 504-12.
3. Ross JDC, Wilson J, Workowski KA, et al. Oral gepotidacin for the treatment of uncomplicated urogenital gonorrhoea (EAGLE-1): a phase 3 randomised, open-label, non-inferiority, multicentre study. *Lancet* 2025; **405:** 1608–20.
4. Basarab GS, Kern GH, McNulty J, et al. Responding to the challenge of untreatable gonorrhea: ETX0914, a first-in-class agent with a distinct mechanism-of-action against bacterial Type II topoisomerases. *Sci Rep* 2015; **5**: 11827.
5. Collins JA, Basarab GS, Chibale K, Osheroff N. Interactions between zoliflodacin and *Neisseria gonorrhoeae* gyrase and topoisomerase IV: enzymological basis for cellular targeting. *ACS Infect Dis* 2024; **10**: 3071-82.
6. Alm RA, Lahiri SD, Kutschke A, et al. Characterization of the novel DNA gyrase inhibitor AZD0914: low resistance potential and lack of cross-resistance in *Neisseria gonorrhoeae. Antimicrob Agents Chemother* 2015; **59**: 1478-86.
7. Foerster S, Golparian D, Jacobsson S, et al. Genetic resistance determinants, in vitro time-kill curve analysis and pharmacodynamic functions for the novel topoisomerase II inhibitor ETX0914 (AZD0914) in *Neisseria gonorrhoeae. Front Microbiol* 2015; **6**: 1377.
8. Golparian D, Jacobsson S, Sánchez-Busó L, et al. GyrB in silico mining in 27151 global gonococcal genomes from 1928-2021 combined with zoliflodacin in vitro testing of 71 international gonococcal isolates with different GyrB, ParC and ParE substitutions confirms high susceptibility. *J Antimicrob Chemother* 2022; **78**: 150-4.
9. Jacobsson S, Golparian D, Oxelbark J, et al. Pharmacodynamic evaluation of dosing, bacterial kill, and resistance suppression for zoliflodacin against *Neisseria gonorrhoeae* in a dynamic hollow fiber infection model. *Front Pharmacol* 2021; **12**: 682135.
10. Jacobsson S, Golparian D, Oxelbark J, et al. Pharmacodynamic evaluation of zoliflodacin treatment of *Neisseria gonorrhoeae* strains with amino acid substitutions in the zoliflodacin target GyrB using a dynamic hollow fiber infection model. *Front Pharmacol* 2022; **13**: 874176.
11. Jacobsson S, Golparian D, Oxelbark J, et al. Pharmacodynamics of zoliflodacin plus doxycycline combination therapy against *Neisseria gonorrhoeae* in a gonococcal hollow-fiber infection model. *Front Pharmacol* 2023; **14**: 1291885.
12. Golparian D, Jacobsson S, Ohnishi M, Unemo M. Complete reference genome sequence of the clinical *Neisseria gonorrhoeae* strain H035, with resistance to the novel antimicrobial zoliflodacin, identified in Japan in 2000. *Microbiol Resour Announc* 2023; **12**: e0113022.
13. Jacobsson S, Cherdtrakulkiat T, Golparian D, et al. High susceptibility to the novel antimicrobial zoliflodacin among *Neisseria gonorrhoeae* isolates in eight WHO Enhanced Gonococcal Antimicrobial Surveillance Programme countries in three WHO regions, 2021-2024. *IJID Reg*. 2025; **15**: 100624.
14. Jacobsson S, Golparian D, Alm RA, et al. High in vitro activity of the novel spiropyrimidinetrione AZD0914, a DNA gyrase inhibitor, against multidrug-resistant *Neisseria gonorrhoeae* isolates suggests a new effective option for oral treatment of gonorrhea. *Antimicrob Agents Chemother* 2014; **58**: 5585-8.
15. Unemo M, Ahlstrand J, Sánchez-Busó L, et al. High susceptibility to zoliflodacin and conserved target (GyrB) for zoliflodacin among 1209 consecutive clinical *Neisseria*

gonorrhoeae isolates from 25 European countries, 2018. *J Antimicrob Chemother* 2021; **76**: 1221-8.
16. Bradford PA, Miller AA, O'Donnell J, Mueller JP. Zoliflodacin: an oral spiropyrimidinetrione antibiotic for the treatment of *Neisseria gonorrheae*, including multi-drug-resistant isolates. *ACS Infect Dis* 2020: **6**: 1332-45.
17. Unemo M, Ringlander J, Wiggins C, Fredlund H, Jacobsson S, Cole M. High in vitro susceptibility to the novel spiropyrimidinetrione ETX0914 (AZD0914) among 873 contemporary clinical *Neisseria gonorrhoeae* isolates from 21 European countries from 2012 to 2014. *Antimicrob Agents Chemother* 2015; **59**: 5220-5.
18. Jacobsson S, Kularatne R, Kittiyaowamarn R, et al. High *in vitro* susceptibility to the first-in-class spiropyrimidinetrione zoliflodacin among consecutive clinical *Neisseria gonorrhoeae* isolates from Thailand (2018) and South Africa (2015-2017). *Antimicrob Agents Chemother* 2019; **63**: e01479-19.
19. Le W, Su X, Lou X, et al. Susceptibility trends of zoliflodacin against multidrug-resistant *Neisseria gonorrhoeae* clinical isolates in Nanjing, China, 2014 to 2018. *Antimicrob Agents Chemother* 2021; **65**: e00863-20.
20. Papp JR, Lawrence K, Sharpe S, Mueller J, Kirkcaldy RD. In vitro growth of multidrug-resistant *Neisseria gonorrhoeae* isolates is inhibited by ETX0914, a novel spiropyrimidinetrione. *Int J Antimicrob Agents* 2016; **48**: 328-30.
21. Su XH, Wang BX, Le WJ, et al. Multidrug-resistant *Neisseria gonorrhoeae* isolates from Nanjing, China, are sensitive to killing by a novel DNA gyrase inhibitor, ETX0914 (AZD0914). *Antimicrob Agents Chemother* 2015; **60**: 621-3.
22. Gibson EG, Bax B, Chan PF, Osheroff N. Mechanistic and structural basis for the actions of the antibacterial gepotidacin against *Staphylococcus aureus* gyrase. *ACS Infect Dis* 2019; **5**: 570-81.
23. Watkins RR, Thapaliya D, Lemonovich TL, Bonomo RA. Gepotidacin: a novel, oral, 'first-in-class' triazaacenaphthylene antibiotic for the treatment of uncomplicated urinary tract infections and urogenital gonorrhoea. *J Antimicrob Chemother* 2023; **78**: 1137-42.
24. Jacobsson S, Golparian D, Scangarella-Oman N, Unemo M. In vitro activity of the novel triazaacenaphthylene gepotidacin (GSK2140944) against MDR *Neisseria gonorrhoeae. J Antimicrob Chemother* 2018; **73**: 2072-7.
25. Scangarella-Oman NE, Hossain M, Dixon PB, et al. Microbiological analysis from a phase 2 randomized study in adults evaluating single oral doses of gepotidacin in the treatment of uncomplicated urogenital gonorrhea caused by *Neisseria gonorrhoeae. Antimicrob Agents Chemother* 2018; **62**: e01221-18.
26. David A, Golparian D, Jacobsson S, et al. In silico gepotidacin target mining among 33213 global *Neisseria gonorrhoeae* genomes from 1928 to 2023 combined with gepotidacin MIC testing of 22 gonococcal isolates with different GyrA and ParC substitutions. *J Antimicrob Chemother* 2024; **79**: 2221-6.
27. VanScoy BD, Scangarella-Oman NE, Fikes S, et al. Relationship between gepotidacin exposure and prevention of on-therapy resistance amplification in a *Neisseria gonorrhoeae* hollow-fiber *in vitro* infection model. *Antimicrob Agents Chemother* 2020; **64**: e00521-20.
